# Localization of the sinoatrial and atrioventricular nodal region in neonatal and juvenile ovine hearts

**DOI:** 10.1371/journal.pone.0232618

**Published:** 2020-05-07

**Authors:** Jordan K. Johnson, Brian K. Cottle, Abhijit Mondal, Robert Hitchcock, Aditya K. Kaza, Frank B. Sachse

**Affiliations:** 1 Department of Biomedical Engineering, University of Utah, Salt Lake City, Utah, United States of America; 2 Nora Eccles Harrison Cardiovascular Research and Training Institute, Salt Lake City, Utah, United States of America; 3 Cardiac Surgery, Boston Children’s Hospital and Harvard Medical School, Boston, Massachusetts, United States of America; University at Buffalo - The State University of New York, UNITED STATES

## Abstract

Localization of the components of the cardiac conduction system (CCS) is essential for many therapeutic procedures in cardiac surgery and interventional cardiology. While histological studies provided fundamental insights into CCS localization, this information is incomplete and difficult to translate to aid in intraprocedural localization. To advance our understanding of CCS localization, we set out to establish a framework for quantifying nodal region morphology. Using this framework, we quantitatively analyzed the sinoatrial node (SAN) and atrioventricular node (AVN) in ovine with postmenstrual age ranging from 4.4 to 58.3 months. In particular, we studied the SAN and AVN in relation to the epicardial and endocardial surfaces, respectively. Using anatomical landmarks, we excised the nodes and adjacent tissues, sectioned those at a thickness of 4 μm at 100 μm intervals, and applied Masson’s trichrome stain to the sections. These sections were then imaged, segmented to identify nodal tissue, and analyzed to quantify nodal depth and superficial tissue composition. The minimal SAN depth ranged between 20 and 926 μm. AVN minimal depth ranged between 59 and 1192 μm in the AVN extension region, 49 and 980 μm for the compact node, and 148 and 888 μm for the transition to His Bundle region. Using a logarithmic regression model, we found that minimal depth increased logarithmically with age for the AVN (R^2^ = 0.818, P = 0.002). Also, the myocardial overlay of the AVN was heterogeneous within different regions and decreased with increasing age. Age associated alterations of SAN minimal depth were insignificant. Our study presents examples of characteristic tissue patterns superficial to the AVN and within the SAN. We suggest that the presented framework provides quantitative information for CCS localization. Our studies indicate that procedural methods and localization approaches in regions near the AVN should account for the age of patients in cardiac surgery and interventional cardiology.

## Introduction

In the mammalian heart, the cardiac conduction system (CCS) is responsible for initiation and propagation of electrical signals that trigger and synchronize mechanical function [1, 2]. The CCS consists of the sinoatrial node (SAN), the atrioventricular node (AVN), the His bundle, the left and right bundle branches, and the Purkinje fiber network. Dysfunction of the CCS is associated with high morbidity and mortality [3–7] and has also been linked to myocardial ischemia and infarction, cardiotoxicity of drugs, and complications due to surgical and interventional procedures [8–14]. Various surgical and interventional procedures can lead to CCS dysfunction including reconstructive surgeries for repair of critical congenital heart defects, ablation procedures in the right atrium (RA), and valve replacement [15, 16]. Avoidance of trauma which can potentially disrupt CCS conduction is crucial to the procedures. While adverse effects of these procedures were reduced over the years, procedural complications due to poor CCS localization are still prevalent [17–22]. Beyond the importance of accurate localization of the CCS in surgical procedures, insights into the arrangement of the CCS are of high clinical relevance for other cardiac procedures such as His bundle ablation for treatment of supraventricular arrhythmia and His bundle pacing [23, 24]. In contrast to right ventricular pacing, His bundle pacing yields a more synchronous activation of the ventricles. Success rate of the pacing approach is highly dependent on exact lead placement, which is, in-turn dependent on accurate identification of His bundle location.

Histological studies extensively characterized the morphology of CCS components and their relation to superficial anatomical landmarks [25–29]. New approaches to identify details of the CCS provided additional insights into the locations and distribution of the CCS [30–32]. Despite these characterizations, it remains difficult to localize the CCS during clinical procedures [33–35]. For surgical procedures, the localization of CCS components is currently based on identification of superficial anatomical landmarks. Though these landmarks provide a generalized location of CCS components, the markers provide incomplete information on the precise distribution of the complex microstructure of the CCS. The complex microstructure includes regions with different CCS cell types, e.g. pacemaker (P) and transition cells in SAN tissue [36, 37] as well as the primary body and peripheral regions of the SAN, and extensions and compact nodal region of the AVN [38]. In addition, the locations of these components and their structures vary in individuals depending on many factors including age and disease including congenital heart defects [33, 39].

Intraoperative imaging and probing technologies for localization of the CCS have been developed since the early 1960s [40, 41]. However, many of these technologies did not yield the sensitivity and specificity necessary to positively impact the outcome of clinical procedures. Additionally, some of these technologies do not integrate well into the workflow in the operating room. Recent work in this field including computed tomography and fiber-optic confocal microscopy (FCM) have shown promise in intraoperative localizing the CCS [32, 42–44]. CCS localization based on computed tomography requires changes to procedural setups and relies heavily on anatomical landmarks near the CCS. FCM allows physicians to acquire real-time images of the SAN and AVN in beating or arrested hearts. FCM has been shown to be safe and easily integrated into the operating space, but it has yet to be assessed for localization of CCS in clinical studies [31, 42, 43, 45–47].

One step in advancing intraoperative localization of the CCS and effectiveness of cardiac procedures is to establish a reliable geometric characterization of the cardiac anatomy within the vicinity of CCS components. Many studies have presented insights into the localization and composition of the CCS [30, 31, 48–51], but this information is commonly difficult to translate for intraoperative localization. Furthermore, information on age-dependent and disease-associated changes of CCS location is sparse.

Here, we quantitatively analyzed the SAN and AVN regions in neonatal and juvenile ovine hearts. We investigated the SAN region including the epicardial surface of the RA and superior vena cava (SVC) junction, and paranodal regions in the proximity of the crista terminalis. The studied AVN region included the RA endocardial surface that covers AVN origination, AVN transition to His bundle, His bundle, and start of the left and right bundle branches. We utilized Masson’s trichrome staining, high resolution imaging and methods of digital image analysis to serially track and characterize the SAN and AVN. Our analysis provides information on the spatial location of the SAN and AVN, and their variability. We quantified depth of the nodes in relation to postmenstrual heart age using logarithmic regression analysis. Furthermore, we assessed tissue composition in nodal tissue regions and established its relationship to age. The presented approach provides a framework for processing of high-quality serial images and a methodology for CCS localization in various species including human.

## Results

**Fig 1:**
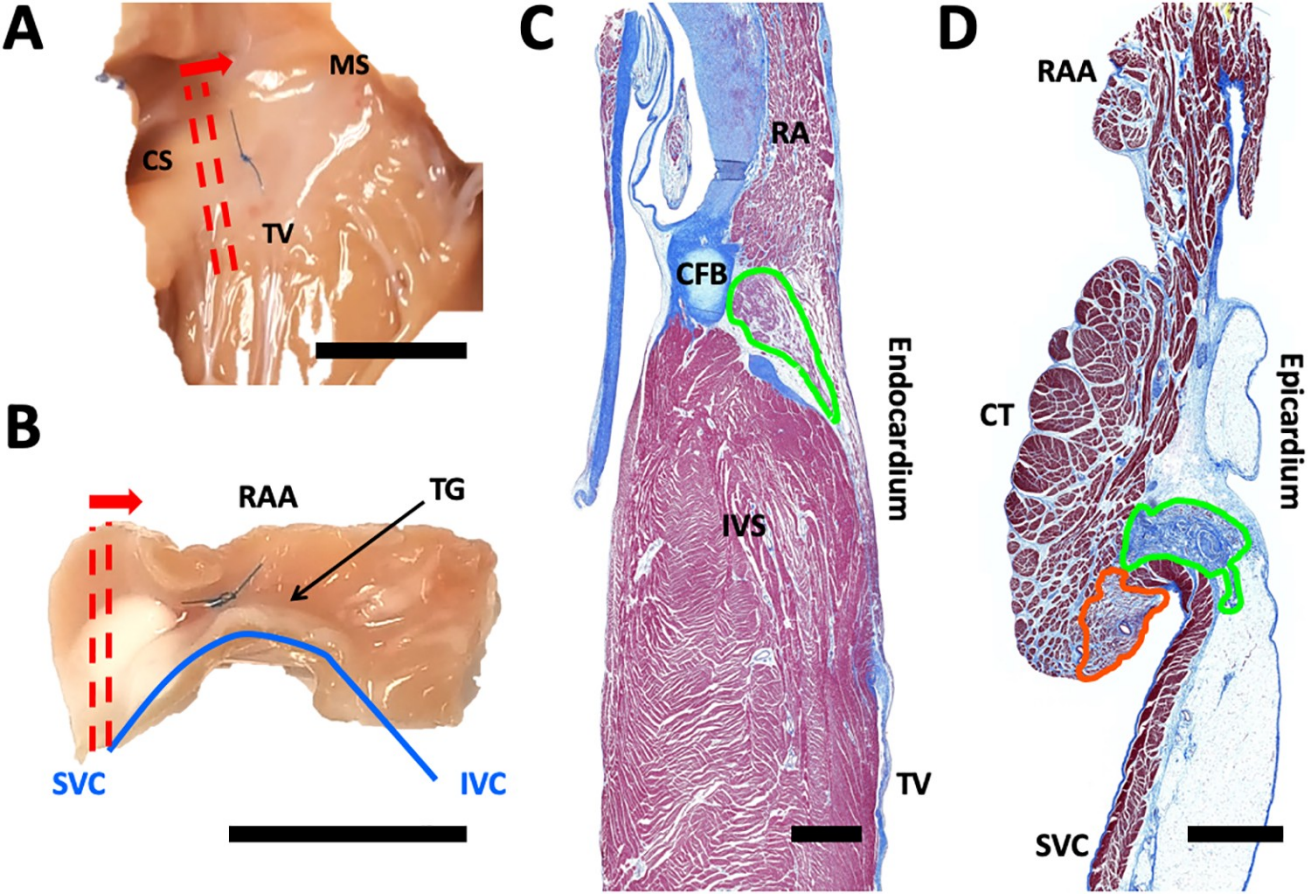
Sectioning and Masson’s trichrome imaging of SAN and AVN. (A) Endocardial view on ovine AVN and (B) epicardial view on SAN region with sectioning orientation and direction indicated by red lines and arrow, respectively. Sutures in (A) and (B) represent approximate node locations identified using anatomical landmarks before heart excision. Scale bars: 1 cm. Example Masson’s trichrome stained sections from (C) AVN and (D) SAN region. Nodes are outlined in green. SAN paranodal region outlined in orange. Scale bars: 1 mm. CS, coronary sinus; MS, membranous septum; TV, septal leaflet of the tricuspid valve; RAA, right atrial appendage; TG, terminal groove; SVC, superior vena cava; IVC, inferior vena cava; RA, right atrium; CFB, central fibrous body; IVS, interventricular septum; CT, crista terminalis.

We analyzed serial sections from AVN and SAN regions of 8 ovine hearts. Sectioning of AVN regions started at the coronary sinus (CS) and ended close to the membranous septum (MS) (Fig 1A). Sectioning of the SAN region started at the SVC-RA junction and ended near the inferior vena cava (IVC)-RA junction (Fig 1B). An example Masson’s trichrome image of an AVN section is shown in Fig 1C. In this image, the node resides superficial to the central fibrous body and follows the contours of the interventricular septum (IVS) along with the RA overlay below the RA endocardial surface. We present an example Masson’s trichrome image of a SAN section in Fig 1D. The SAN is located on the SVC side of the crista terminalis on the endocardial side and terminal groove (TG) on the epicardial side. The node often resides beneath a fat pad and in close proximity to the paranodal region.

**Fig 2:**
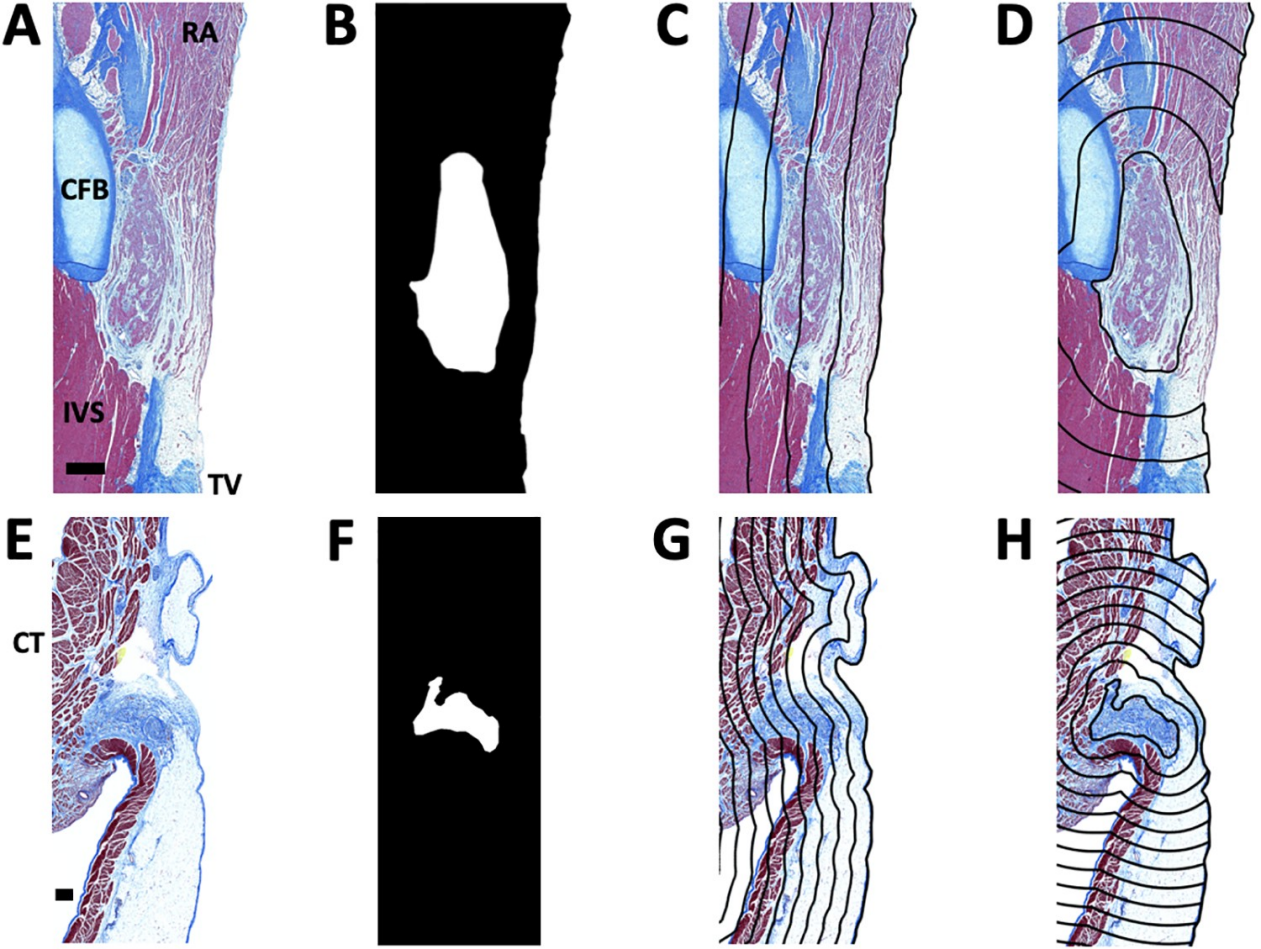
Analyses of Masson’s trichrome images. (A-D) Segmentation process for AVN. (A) Cropped transverse section of AVN region. Scale bar: 500 μm. (B) Segmentation of endocardial surface and AVN, indicated in white. Contour plot of distance map from (C) endocardial surface and node (D) with line spacing of 500 μm overlaid on (A). (E-H) Segmentation process for SAN. (E) Cropped transverse section of SAN region. Scale bar: 200 μm. (F) Segmentation of epicardial surface and SAN, indicated in white. Contour plot of distance map from (G) epicardial surface and (H) node with line spacing of 200 μm overlaid on (E). RA, right atrium; CFB, central fibrous body; CT, crista terminalis.

The number of sections per AVN and SAN ranged from 11 to 24 and 13 to 60, respectively. After cropping, we segmented nodal regions and the tissue surface (Fig 2). The tissue surface segmentation was fit to a spline function. We calculated the distance from the tissue surface as well as the SVN (Fig 2C and 2D) and AVN (Fig 2G and 2H) within the tissue.

**Fig 3:**
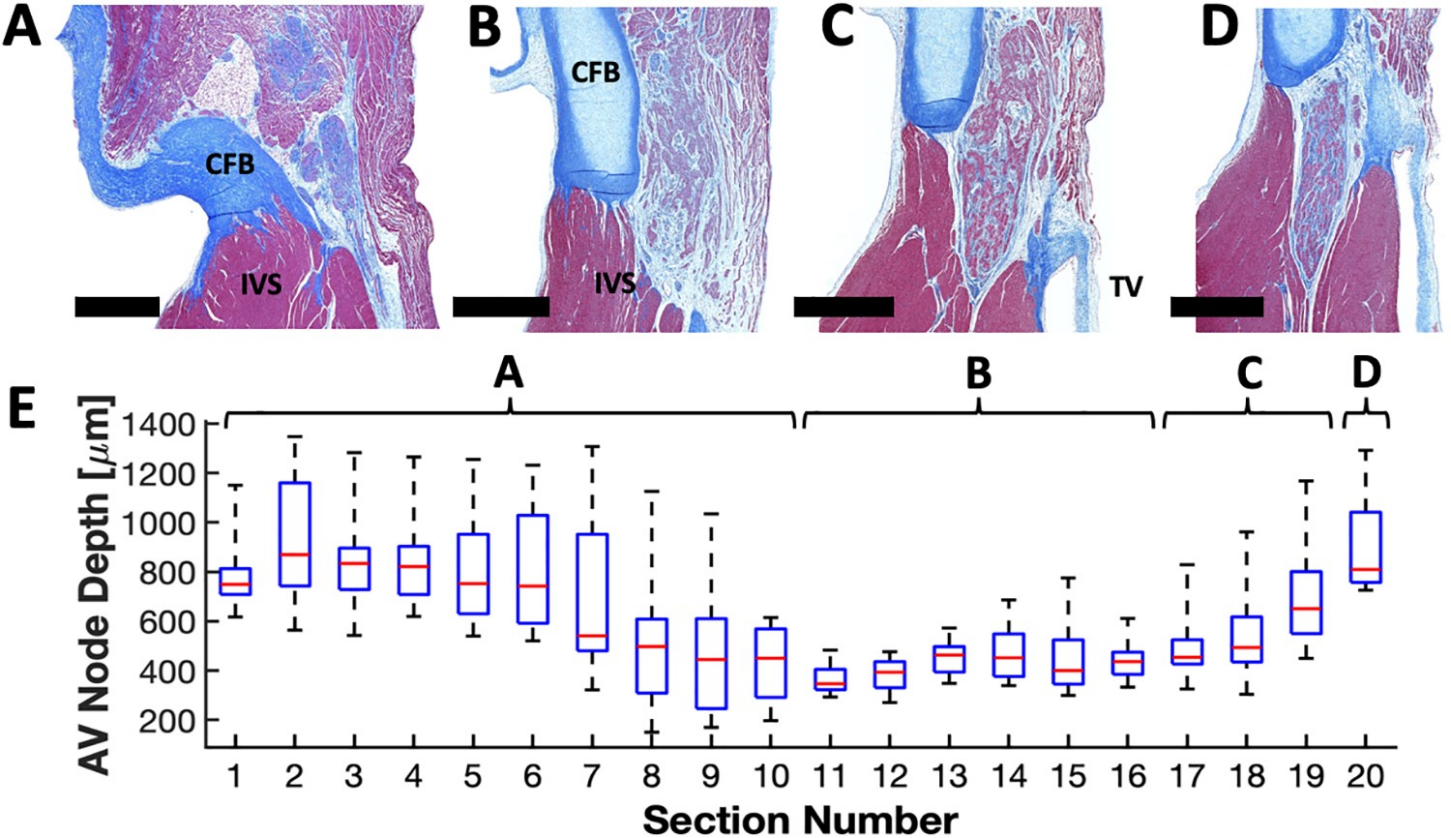
Masson’s trichrome images and depth analyses from 4 different anatomical regions of the AVN-His bundle complex. (A) Cropped transverse section of AVN extension region with separation of left and right AVN extensions. (B) Cropped transverse section of AVN compact node region. (C) Cropped transverse section of AVN transition to His bundle region. (D) Cropped transverse section of His bundle. (E) Depth profile of AVN from RA endocardial surface. Box plots represent the range of depths for each section along the projection of the node onto the endocardial surface. Sections were collected every 200 μm. Scale bars: 500 μm. CFB, central fibrous body; IVS, interventricular septum; TV, septal leaflet of the tricuspid valve.

An overview of sections from an example AVN is shown in Fig 3A–3D. Nodal depth and coverage analysis were aided by dividing the sections into 4 anatomical regions, i.e. the AVN extension region (Fig 3A), compact node region (Fig 3B), transition to His bundle region (Fig 3C), and the His bundle and distal components of the CCS (Fig 3D). The left and right AVN extensions (Fig 3A) combined to form the compact AVN. The compact node (Fig 3B) penetrated the connective tissue connecting the central fibrous body to the interventricular septum (Fig 3C). This transition of the AVN ended when the His bundle is fully formed and distal from atrial tissue (Fig 3D). We summarize measurements of the depth of the AVN, i.e. the distance from endocardial surface to nodal surface, in Fig 3E. The depth of this AVN varied widely reaching a minimum of 150 μm at section #8, and a maximum of 1348 μm at section #2 (Fig 3E).

**Fig 4:**
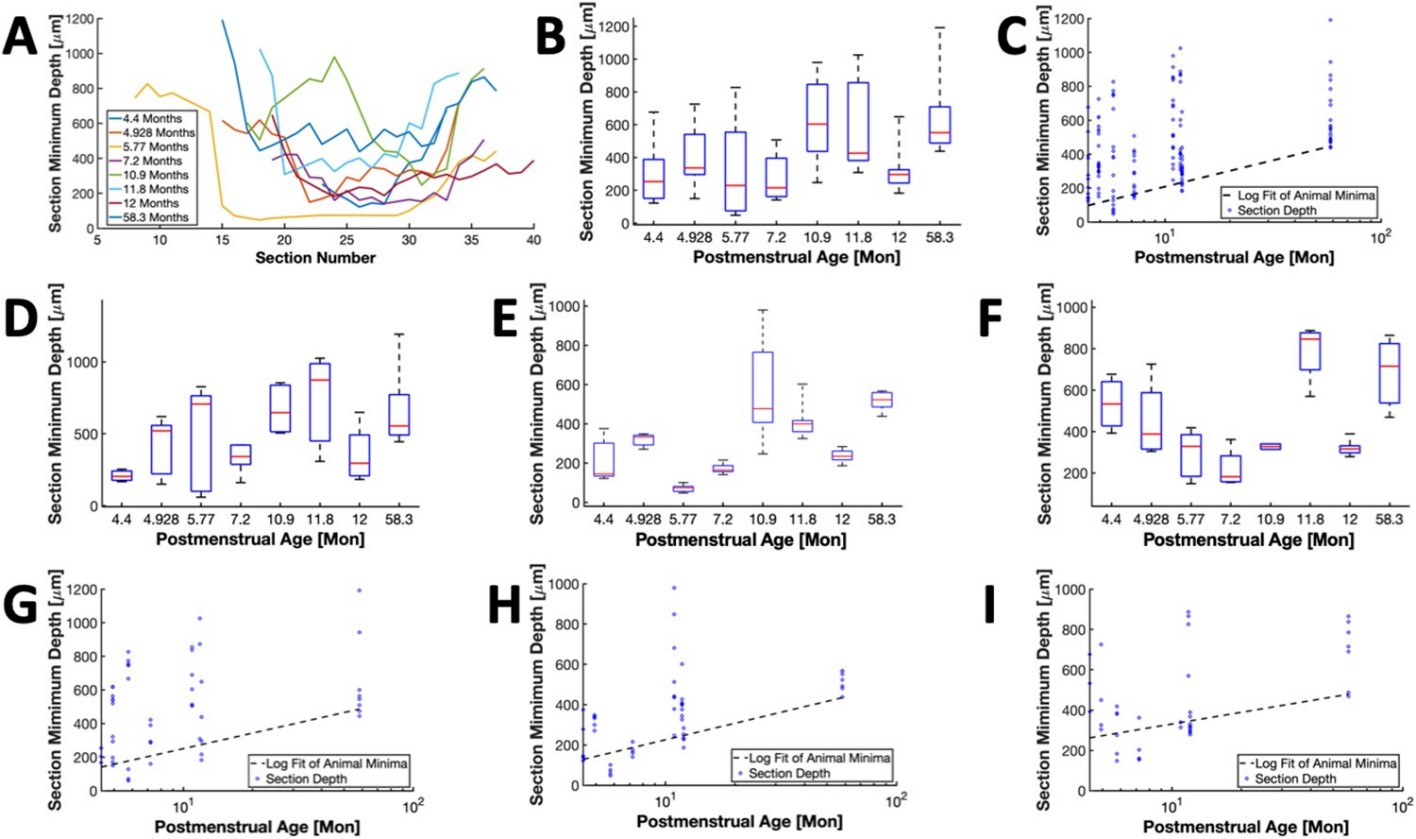
Depth analyses of AVN-His bundle complex. (A) Aligned AVN depth profiles from the RA endocardial surface for 8 hearts. Postmenstrual age of each animal is shown in the key. (B) Box plot of AVN minimal depths for each section. (C) Scatter plot for minimal depths. Logarithmic regression modeling revealed a strong relationship of minimal AVN depth and age (R^2^ = 0.818, P = 0.002). Box plot of minimum depths versus in sections from AVN (D) extension, (E) compact node, and (F) transition to His bundle region for each heart. Logarithmic regression modeling in the AVN (G) extension (P>0.05), (H) compact node (R^2^ = 0.617, P = 0.021), and (I) transition to His bundle region (P>0.05).

We present statistical analyses of the depth of AVNs in Fig 4. Fig 4A provides a summary of aligned AVN minimum depth topology. The transition of AVN to His bundle was used as the alignment origin. Fig 4B details AVN depths across the hearts using box plots indicating depth mean and quartile ranges. Minimal depth was 440±240 μm and ranged between 48 and 1192 μm. Logarithmic regression analysis (Eq 2) on data presented in Fig 4B revealed an increase in nodal minimal depth with age (R^2^ = 0.818, P = 0.002, offset a = -102.607 and rate b = 135.018) (Fig 4C). The high R^2^ suggests that the majority of depth variation is related to age.

Depth analyses of AVN regions are presented in Fig 4D–4I. Fig 4D and 4G show depth in the AVN extension region and a logarithmic regression model for the relationship between depth minima and age, respectively. Only sections before formation of the compact node were considered. The regression analysis of Fig 4G did not yield a significance difference to a constant model (P>0.05). Fig 4E and 4H show the depth and logarithmic regression analysis of the compact node region, respectively. Only sections before the transition to His bundle were applied. The regression analysis demonstrated a strong depth minima-age relationship with (R^2^ = 0.617, and P = 0.021, a = -47.525 and b = 118.613). Fig 4F and 4I detail the depth range and logarithmic regression of the transition to His bundle region. Only sections before the His bundle were considered. Logarithmic regression analysis did not yield a significant difference to a constant model (P>0.05).

**Fig 5:**
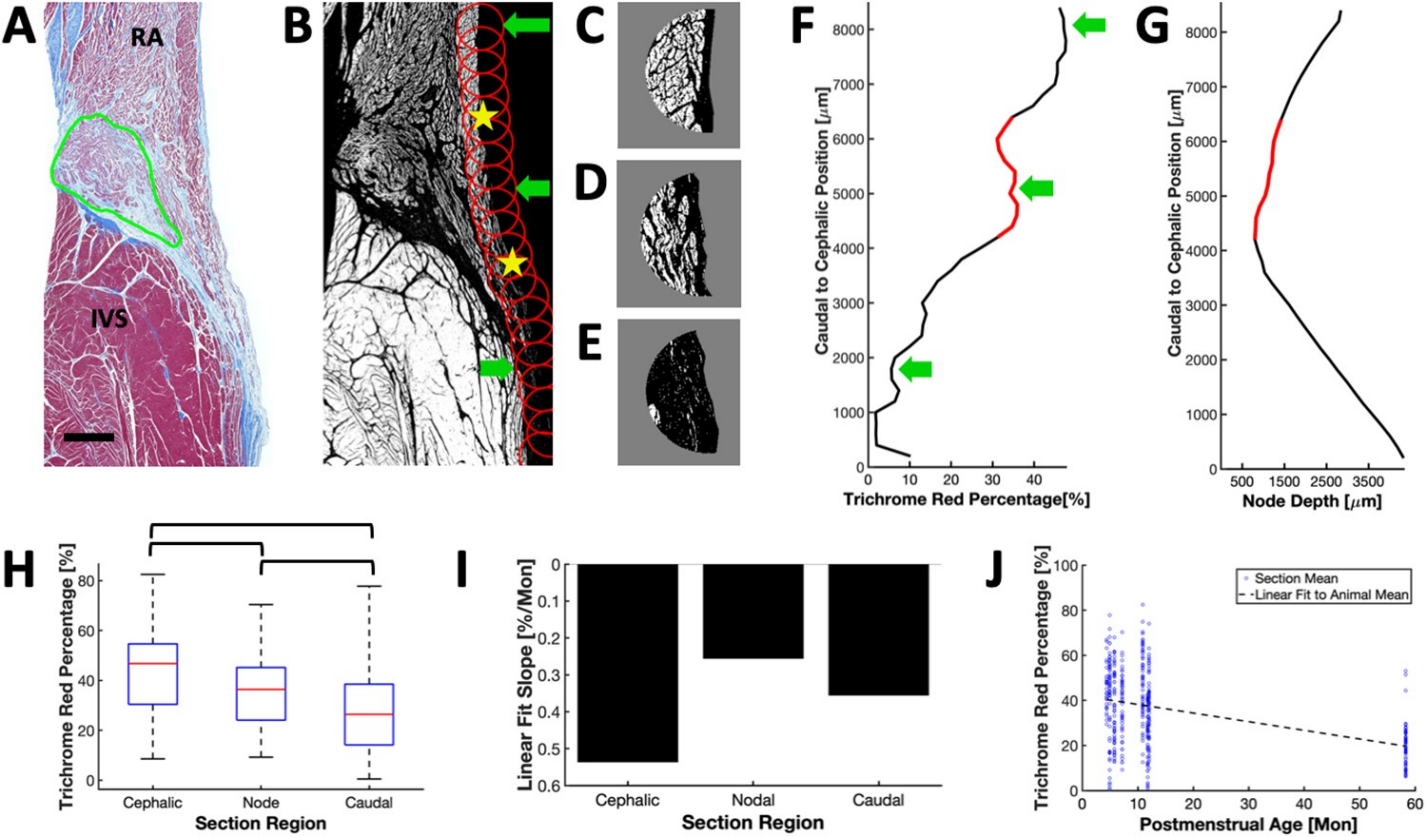
Coverage analyses of AVN. (A) Masson’s trichrome transverse section with outlined compact node. Scale bar: 1 mm. (B) Red threshold image of (A) with representative ROIs plotted at half the original density. Yellow stars indicate borders along the endocardial surface between the nodal, cephalic (top) and caudal (bottom) regions of the endocardial surface. Green arrows indicate ROIs displayed in (C-E). (C-E) Red threshold image in ROI corresponding to the top, middle and bottom green arrow, respectively in (B). Gray areas indicate regions not part of the analysis. (F) AVN coverage of Masson’s trichrome red percentage from each of the circular ROI mapped to the section surface. Green arrows indicate the ROI depicted in (C-E). (G) Depth of node beneath the endocardial surface. Red line segments in (F) and (G) mark the nodal region. (H) Trichrome red percentage for AVN surface regions divided into cephalic, nodal and caudal. Red percentages are different in-between groups (cephalic:nodal P = <0.001, cephalic:caudal P = <0.001, nodal:caudal P = <0.001). (I) Slope from linear regression analysis of trichrome red percentage with respect to age for all animals and sections. Red percentage decreased with age. (J) Linear regression of mean red percentages revealed a decreased myocardial overlay with increasing age (R^2^ = 0.510; P = 0.047).

We analyzed the peripheral and superficial tissues in Masson’s trichrome sections of AVN and SAN to characterize tissue coverage of the nodes. Endocardial tissue surfaces in AVN sections, such as the example in Fig 5A, were divided into 3 section regions, i.e. the nodal region or nodal projection region as well as the cephalic and caudal regions adjacent to the nodal region (Fig 5B). Dual red thresholding and mapping of circular regions of interest (ROI) allowed for quantification of the trichrome red percentage along the length of tissue surface (Fig 5B). We used the trichrome red percentage as a measure of myocardial tissue content. Example ROIs with their trichrome red percentage are shown in Fig 5C–5E. Comparison of trichrome red percentages and nodal depth are presented in Fig 5F and 5G. The trichrome red percentage decreased from the cephalic to the caudal regions (Fig 5H). In this example, the superficial cephalic and nodal regions exhibited a pronounced overlay with atrial myocardium. The red percentage was different between all three regions (cephalic:nodal P<0.001, cephalic:caudal P<0.001, nodal:caudal P<0.001). Linear regression analysis (Eq 1) yielded a negative relationship between the red percentage and age in each region (Fig 5I) suggesting a decrease of myocardial overlay with increasing age. Linear regression analysis (Eq 1) of the mean red trichrome percentage for all animals (Fig 5J) revealed a total decrease in trichrome red percentage of over 10% across the age span of the hearts (R^2^ = 0.510, P = 0.047, a = 42.039, b = 0.383).

**Fig 6:**
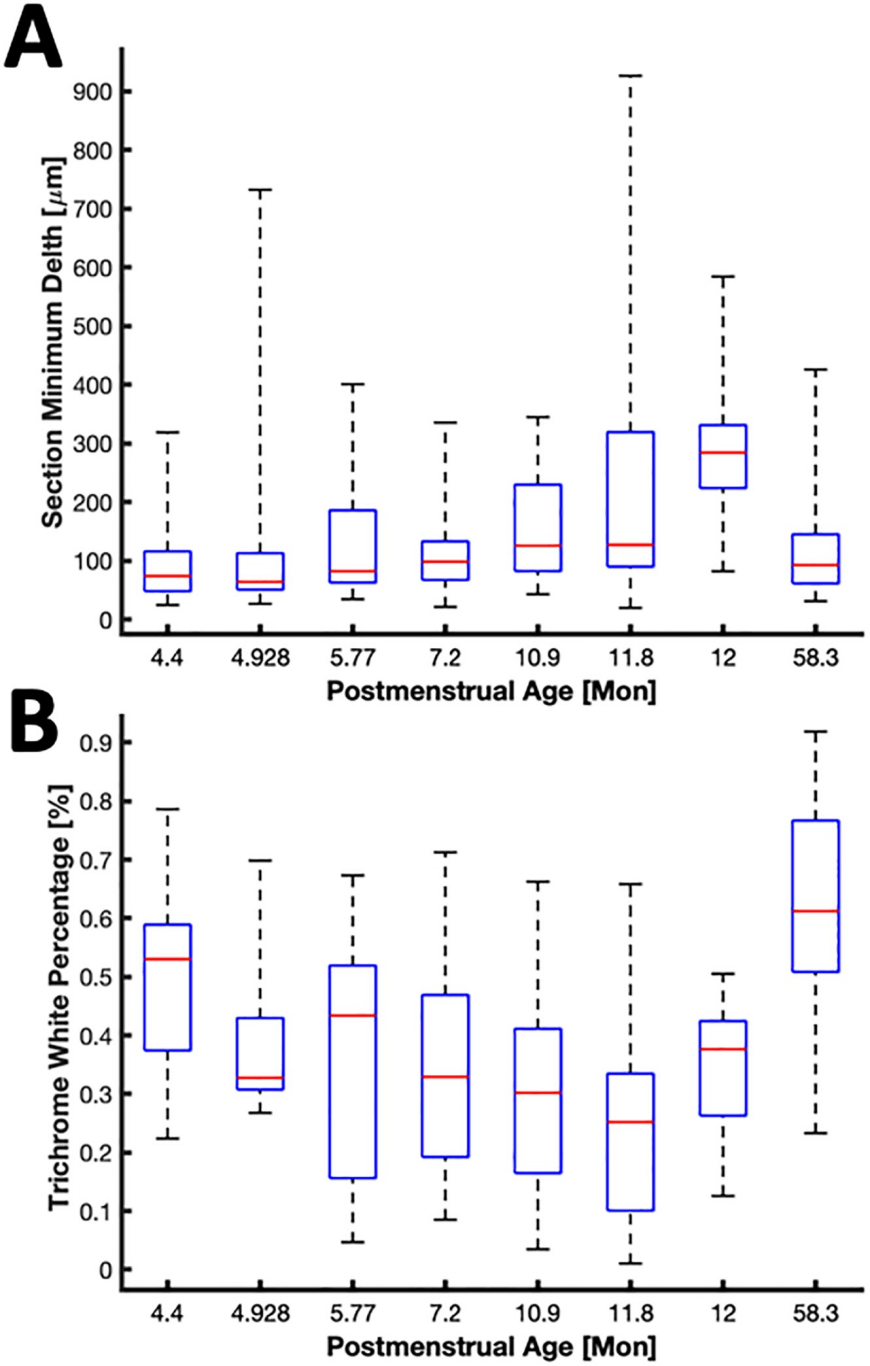
Depth and coverage analyses of SAN. (A) Box plot of minimal depths of sections versus age. (B) Box plot of trichrome white percentage from SAN ROI versus age. A logarithmic model of the relationship between age and SAN features was not statistically different from a constant model.

SAN depth analyses followed a similar process as AVN sections. Minimal depths for SAN sections across the 8 animals are shown in Fig 6A. Minimal depth was 135±116 μm and ranged between 20 and 926 μm. Linear and logarithmic regression models of the age-depth relationship did not yield a significant different versus a constant model (P>0.05). In contrast to the measurement of trichrome red percentage for myocardial overlay quantification in AVN sections, we analyzed trichrome white coverage of the SAN sections to identify overlay by adipose tissue. Trichrome white percentages for ROIs in sections from each animal were highly variable (43±20%) and ranged between 1 and 92% (Fig 6B). Linear and logarithmic regression models of the relationship between SAN depth and trichrome white coverage did not yield a significant difference versus a constant model (P>0.05).

**Fig 7:**
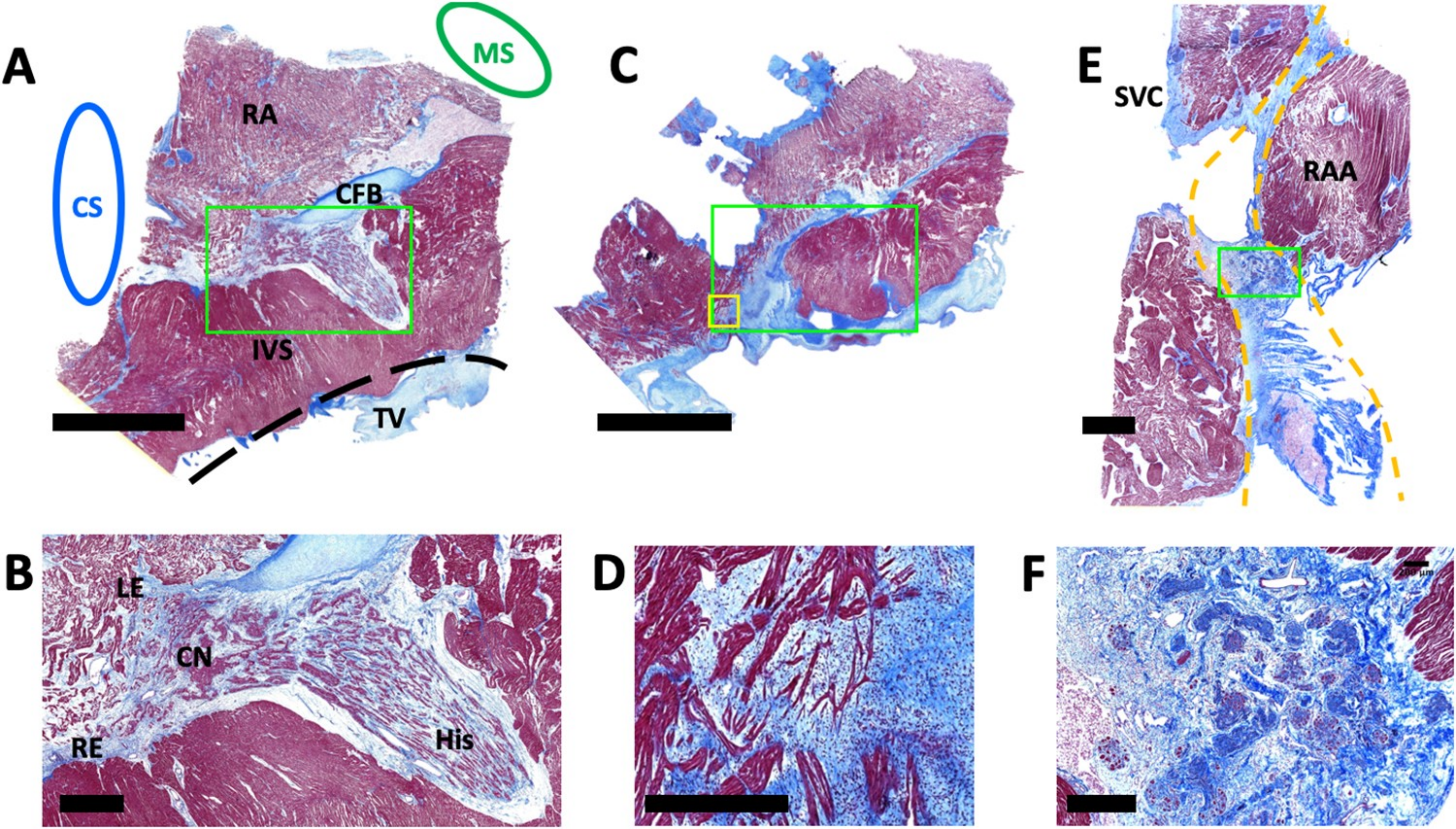
Example Masson’s trichrome sections of AVN and SAN. (A) AVN section parallel to the endocardial surface at a depth of approximately 300 μm. Scale bar: 5 mm. (B) Zoomed region from green box in (A) with left and right AVN extensions, compact node, and the His bundle. Scale bar: 500 μm. (C) AVN section parallel to endocardial surface at a depth of 50 μm showing the terminal region of the RA overlay. Location of green boxes in (A) and (C) were matched. Scale bar: 5 mm. (D) Zoomed region from yellow box in (C) with reticulated cells of the RA overlay. (E) SAN section parallel to epicardial surface at a depth of approximately 100 μm with terminal groove and node. Yellow lines mark boundary of terminal groove. Scale bar: 2 mm. (F) Zoomed region of from green box in (E). Scale bar: 500 μm. CS, coronary sinus; RA, right atrium; MS, membranous septum; CFB, central fibrous body; IVS, interventricular septum; TV, septal leaflet of the tricuspid valve; LE, left nodal extension; RE, right nodal extension; CN, compact node; His, His bundle; SVC, superior vena cava; RAA, right atrial appendage.

To complement our quantification of nodal regions from images from transverse sectioning, we performed sectioning parallel to the tissue surface. Example sections from this method are shown in Fig 7. This mode of sectioning allowed for assessment of nodal depth and coverage analyses previously performed, and yielded images of CCS components in a transmural sequence. Fig 7A illustrates the 4 regions of the AVN and His bundle complex previously shown in Fig 3A–3D at a depth of approximately 300 μm. The tissue morphology of each region of the AVN is also shown in greater detail in Fig 7B. Visualization of the area superficial to the AVN and His bundle structure revealed reticulated RA overlay cells that lie directly on top of the AVN extension and compact node regions (Fig 7C and 7D). Surface parallel sectioning of the SAN region is presented in Fig 7E. The morphology of the SAN region (Fig 7F) matches segmented SAN tissue in transverse sections (Fig 1D).

## Discussion

We introduced methods for localization of the CCS and studying age-associated alterations of the relationship of CCS components and tissue surface. We applied AVN and SAN samples from 8 ovine hearts for depth and morphology analyses on serial Masson’s trichrome sections. We analyzed 170 AVN sections and 490 SAN sections from animals at different ages. Our studies provided novel information on the nodal depth and superficial tissue composition as well as their modulation by age. Age affected the location of the AVN and its components, but not the SAN.

Using guidelines for identification of CCS microstructure established over the past century [30, 34, 52–55], we segmented high resolution images collected every 100 μm through AVNs and SANs (Fig 2). Transverse serial sectioning of AVN tissue confirmed the existence of the left and right AVN extensions in the ovine heart, similar as in human [38]. Also, we observed distinct microstructural changes from the compact node region to the His bundle (Figs 3C vs. 7B).

The analyses yielded a similar depth distribution from the AVN extensions to His bundle across all ages (Fig 4A). The MS side of the AVN extensions and their fusion into compact node body were the most superficial portion of the AVN (Figs 3E, 4D and 4E). Compared to those sites, the origination of the AVN extensions and the transition to His bundle region exhibited increased depth (Figs 3E and 4F). These findings indicate that surgical interventions in AVN regions should account for local AVN depth to avoid damage of the CCS.

Our work revealed that minimum AVN depth increases logarithmically with age (Fig 4). This increase in depth occurred for the complete node (Fig 4C) and in the compact region. Regression analysis of the relationship between age and depth in the extension and His bundle region was inconclusive. Our findings suggest that procedural methods and localization approaches in cardiac surgery and interventional cardiology should be adapted to the age of patients. We consider three fields of application of our findings: surgical tissue handling, placement and design of pacing electrodes, and choice of imaging modality and image settings for localization of the AVN. Handling of tissues in surgical procedures should take the age-dependent proximity of the AVN to the endocardial surface into account. In particular the AVN in juveniles is superficial and surgical interventions causing tissue damage and tension at the endocardial surface can be expected to directly affect the AVN. Also, we propose that information on the depth of the AVN is useful for placement of electrical leads and design of pacing electrodes to specifically target this CCS component in the developing heart. Furthermore, our findings suggest that the choice of imaging modalities and image settings should reflect how age affects the location of CCS components. The measured depths of AVNs suggest that imaging techniques require an imaging depth of over 1 mm in order to comprehensively cover the AVN from origination to transition to His bundle. There are, however, sites of minimum depth in each AVN of neonate and juvenile animals that require a smaller imaging depth (<400 μm) for localization.

Our study indicated, through the quantification of trichrome red percentages in tissue superficial to the AVN and imaging of sections parallel to the endocardial surface, that it is possible to use superficial tissue composition and microstructure to identify nodal components. Superficial tissues exhibited significantly different trichrome red percentages between tissue cephalic or caudal to the AVN and the actual AVN nodal region (Fig 5). Our dual red thresholding method allowed us to relate the trichrome red percentage to the local myocardial volume fraction within these complex tissue sections. By detecting variations in myocardial volume fraction around the triangle of Koch, clinicians and researchers will be able to accurately localize deeper nodal structures. The decrease in myocardial volume fraction superficial to the AVN is due to the thinning of the RA overlay and higher volume fraction of connective tissue adjacent to the AVN and within networks of AVN transitional cells [56–58]. Accuracy of such a localization method is further enhanced by the significant decrease in myocardial volume fraction caudal to the node (Fig 5H).

In addition to changes in superficial tissue composition, distinct tissue patterns superficial to the node could serve as reliable markers for deeper AVN structures. For instance, irregular tissue patterns mark the termination of the RA overlay (Fig 7C and 7D). We discovered these superficial structures by sectioning AVN tissue parallel to the endocardial surface. The irregular pattern of the RA overlay termination lies superficial to the fusion of the AVN extensions and the compact node. Similar patterns were previously identified by us in the AVN region of an ovine model of open-heart surgery with FCM with an imaging depth of approximately 65 μm [43].

Our depth and coverage analyses were designed to characterize the complex tissue arrangement in the SAN region. The SAN resides near the surface of the epicardium on the SVC side of the terminal groove [35, 52]. Our work revealed that the minimum depth of SAN from the epicardial surface ranges from 20 to 82 μm. We suggest that FCM or other approaches for imaging of these structures will yield images of microstructure similar to that seen in our Masson’s trichrome images in Figs 1D, 2H and 7F. Our regression analyses did not indicate a relationship between depth of the SAN and age (Fig 6A). While superficial portions of the SAN might be visible with the naked eye, comprehensive localization of the SAN will require an imaging depth of approximately 800 μm. In many SAN sections, the node was not limited to a compact structure at the terminal groove. Rather, we observed many projections of the node that can reach up to 5 mm on both sides of the terminal groove.

To characterize coverage of the SAN by adipose tissue, we quantified the Masson’s trichome white percentage in tissue superficial to the node. This analysis showed that trichrome white percentage varies widely from section to section. We did not find a relationship between the SAN coverage and its depth. Multiple factors contributed to the wide range of trichrome white percentage and the poor relationship to depth including the presence of projections and islands of nodal cells [31] that reach beyond the fat pad toward the epicardial surface as well as sections that include little to no fat pad between the node and the epicardial surface.

### Limitations

This work focused on neonatal and juvenile hearts from ovine. In order to extend the age range and scope of this study, we included an adult ovine heart (53.4 months old). Analysis of the relationship between depth and age including the adult ovine further supported a logarithmic regression model. Without inclusion of this adult heart in the analysis, the neonatal and juvenile ovine hearts still followed a statistically significant logarithmic regression (a = -132.04, b = 150.16, R^2^ = 0.565, P = 0.002). Furthermore, differences of trichrome red percentage between the three AVN regions were still significant (cephalic:nodal P<0.001, cephalic:caudal P<0.001, nodal:caudal P<0.001).

Due to the gradual transition of the SAN to the surrounding myocardium we restricted our definition of SAN tissue to P cell clusters and dense concentrations of transition cells [36, 37, 59]. These restrictions enabled a more uniform and reliable segmentation of the SAN. However, they limited the amount of nodal tissue that was included in the analysis. A different definition of nodal tissue that, for example, includes regions with a gradation of transition cells into myocytes or connective tissue would have increased the nodal area analyzed.

Analyses in our study were performed on images from sequential tissue sections. We limited our analyses to sections without artifacts due to shearing, tearing, excessive folding, or non-uniform immersion in staining solutions. Processing and analyses were limited to 2D images from sections collected at a high spatial resolution, i.e. spacing of 100 μm. Alternatively, 3D analyses would be feasible through computational reconstruction of the AVN and SAN tissue.

A limitation of our ovine model is the lack of established and reliable antibodies for specific labeling of the conduction system. We attempted labeling with antibodies for hyperpolarization-activated cyclic nucleotide-gated channel 4 (HCN4) as in our prior work on the rodent conduction system [42]. Despite extensive variation of the labeling protocol, results were not conclusive. Based on prior work demonstrating differential expression of connexins in the conduction system and working myocardium [60], we also explored labeling with antibodies for connexin 40 and 43. However, the labeling did not yield conclusive results due to lack of antibody specificity.

Translatability of our quantitative findings using an ovine model to the human heart is currently unclear. Similar approaches as described by us are required to fill this gap in quantitative understanding of effects of development and aging on the conduction system in the human heart.

## Methods

### Heart collection and sources

Ovine hearts were obtained through protocols approved by the Institutional Animal Care and Usage Committee (IACUC) at Boston Children’s Hospital and the University of Utah. Postmenstrual ages of the animals ranged from 4.4 (pre-term) to 58.3 months. To account for premature births postmenstrual age was used in all calculations. After euthanasia of the animals, hearts were excised and perfused with a cold, zero-calcium Tyrode solution (in mmol/l: 92 NaCl, 11 dextrose, 4.4 KCl, 5 MgCl_2_, 24 HEPES, 20 taurine, 5 creatine, 5 C_3_H_3_NaO_3_, 1 NaH_2_PO_4_, 12.5 NaOH; pH 7.2; ≈10°C). Subsequently, hearts were fixed using the Tyrode solution containing 4% paraformaldehyde via antegrade perfusion with a syringe. The hearts were then immersed in the same solution for up to 24 h before being stored in phosphate buffered saline until dissection.

### Node dissection

SAN and AVN regions were dissected from each heart using anatomical landmarks [35]. Excised SAN regions included the crista terminalis of the RA and ranged from 2–4 mm beyond the crest of the RA appendage in the cephalic direction to the RA-IVC junction. At least 5 mm of tissue was included on both the SVC and RA appendage (RAA) sides of the crista terminalis. Samples were approximately 1 cm wide (SVC to RA appendage across the terminal groove), 3 cm long (SVC-RA junction to IVC-RA junction) and included both the epicardial and endocardial surface of the RA. Excised AVN regions spanned from proximal to the CS ostium to the MS, and cephalic to the tendon of Todaro to below the annulus of the tricuspid valve septal leaflet. The entire triangle of Koch and cephalic portions of the left and right bundle branches were included in the AVN regions.

### Histology section preparation and imaging

Excised tissue samples were marked for identification of the sectioning face with a tissue dye that withstood paraffinization (CDI’s Tissue Marking Dyes, Cancer Diagnostics, Inc). Samples were then dehydrated through a gradient of ethanol and deionized water solutions which ended in 100% ethanol. After dehydration, the tissue was cleared with a gradient of paraffin-miscible organic solvent called Citrisolv and ethanol solutions. Clearing ended with immersion in 100% Citrisolv. The tissue was then immersed in paraffin solutions with decreasing amounts of Citrisolv and placed in a heated vacuum container at 60 °C. Tissue was completely infiltrated by paraffin wax after two immersion steps in 100% paraffin. Times for each step varied based on tissue sample size. Paraffinized tissue was then placed in a paraffin mold and oriented so that the marked sectioning face of the tissue was accessible. Paraffin blocks hardened overnight.

Sectioning was performed on a HistoCore BIOCUT microtome (Leica Biosystems, Wetzlar, Germany). Paraffin blocks were immersed in a solution of ice water and 16.67% cationic softening agents by volume to minimize section tearing. 8–10 serial sections with a thickness of 4 μm were collected every 100 μm throughout each tissue sample. AVN samples were sectioned from CS to MS. SAN samples were sectioned from RA-SVC junction to RA-IVC junction along the crista terminalis. Sections were collected on positively charged tissue adhesion slides and placed on a slide warmer until paraffinized sections were flush across the slide. Completed slides were stored at room temperature. SAN and AVN samples from one ovine heart were also sectioned parallel to the epicardial and endocardial tissue surface, respectively. Half of the collected sections were stained using an automated Masson’s trichrome protocol on an automated staining device (Dako Corporation, Carpinteria, CA). Stain immersion duration and differentiation were verified using liver tissue sections of the same thickness every 16 sections.

Imaging of the serial sections was performed on an AXIOSCAN Z.1 slide scanner (Cark Zeiss AG, Oberkochen, Germany) using a 40x objective as well as automated section identification and focus protocols. Image screening, pre-processing, including color balance adjustment and rotation, were performed using Fiji [61]. For subsequent analyses, only images of tissue sections without tearing and folding artefacts were considered.

### Image segmentation and geometrical analyses

We manually segmented sections to identify nodal tissues and marked the epi/endocardial surface using MATLAB (version 2017a and higher, Mathworks, Natick, MA). The AVN was identified according to position in relation to the IVS, endocardial surface, RA tissue and the central fibrous body (Figs 2A and 3A). We further identified the AVN as a grouping of cells with a lighter shade of red than the surrounding myocardium. Due to the complex nature of the SAN transition to surrounding tissue [62, 63] and the difficulty associated with delineating SAN boundaries using histology [52], we restricted our definition of SAN tissue to P cell clusters and dense concentrations of transitional cells [36, 37]. Projections of transitional cells that were small and removed from the primary nodal body were not included in the segmentation. The segmentations were used to analyze the morphology, depth, and coverage of nodal tissue in each section. The segmented epi/endocardial surface was fitted to a smoothing spline function using the MATLAB fit function. Distance maps were generated to calculate the distance from both the node and surface. Nodal depth was determined as the values of the nodal distance map along the fitted surface function points. Full length nodal depth vectors and a minimum nodal depth were recorded for each section.

Sub-epi/endocardial tissue was analyzed for SAN and AVN regions, respectively. For the SAN, epicardial adipose tissue overlay was measured using a white content threshold on the Masson’s trichrome images. White percentages were recorded in conjunction with SAN depth. AVN coverage with sub-endocardial working myocardium was measured using a red content dual threshold on the Masson’s trichrome images. Coverage of AVN was recorded with nodal depth as the red percentage of tissue superficial to any nodal tissue. Percentages were determined by centering a series of circular ROIs on the fitted surface function. SAN ROIs had a radius of 150 μm and were spaced along the surface every 75 μm. AVN circles had a radius of 500 μm and were spaced along the surface every 250 μm. The trichrome white or red percentage within these ROIs and superficial to the nodal mask was averaged.

### Statistical analyses

Nodal depth and coverage were reported as mean +/- standard deviation. Standard box and whisker plots, depicting the mean, quartile ranges, and outliers, were used to illustrate data from the hearts. Linear and logarithmic regression models were created with the MATLAB functions fitlm and fitnlm. The linear and logarithmic models were defined as:

y(age)=a+bage (1)

y(age)=a+blog(age) (2)

with the free parameters a and b. The models were considered significantly different versus a constant model if F statistics yielded a value of P<0.05. The coefficient of determination R^2^ determined the goodness of fit for the regression models. ANOVA with post hoc Tukey-Kramer test was performed on measures of AVN sections to determine significant differences. P-values above 0.05 were presented as P>0.05, and P-values below 0.001 were presented as P<0.001.
